# Endometrial Intraepithelial Neoplasia, Concurrent Endometrial Cancer and Risk for Pelvic Sentinel Node Metastases

**DOI:** 10.3390/cancers16244215

**Published:** 2024-12-18

**Authors:** Tabayi Hawez, Michele Bollino, Celine Lönnerfors, Jan Persson

**Affiliations:** 1 Division of Gynaecologic Oncologic Surgery, Department of Obstetrics and Gynaecology, Skåne University Hospital Lund, 22185 Lund, Sweden; 2 Department of Clinical Sciences, Obstetrics and Gynaecology, Faculty of Medicine, Lund University, 22185 Lund, Sweden

**Keywords:** endometrial intraepithelial neoplasia, endometrial cancer, sentinel lymph node

## Abstract

Background/objectives: Given the risk of a progression, or an undiagnosed endometrial cancer (EC), the treatment of choice is hysterectomy in women with endometrial intraepithelial neoplasia (EIN). The risk of metastatic disease and whether sentinel node (SLN) biopsy should be performed remains unclear. The primary aim of this prospective study was to determine the overall incidence of concurrent EC and the impact of the diagnostic tool used and the type of endometrial lesion. The secondary aim was to investigate the risk of metastatic SLNs. Methods: Between July 2019 and May 2024, 98 consecutive women with EIN deemed suitable for robotic surgery and SLN dissection were included in the study. Ultrastaging and immunohistochemistry were performed on all SLNs. Results: In total, 47% of women with preoperative EIN had EC on final histology; 13% of these had metastatic SLNs and the overall risk of metastases was 6.3%. Women who obtained their diagnosis by an endometrial biopsy had 65% risk of EC. All women with metastatic SLNs had non-polypoid lesions and five out of six obtained their diagnosis through endometrial biopsy. Conclusions: The overall risk of SLN metastases was 6.3%, all in women with a general endometrial thickening and/or a diagnosis of EIN by office endometrial biopsy, suggesting that SLN detection should be offered particularly to women with EIN who fulfill these preoperative criteria.

## Simple Summary

The accuracy of the preoperative diagnosis, the risk of concurrent endometrial cancer (EC) and the need for nodal assessment in women with endometrial intraepithelial neoplasia (EIN) remains unclear. The aim of the study was to assess the incidence of concurrent EC at final histology and the presence of metastases in sentinel lymph nodes (SLNs), overall and related to mode of diagnosis and type of endometrial lesion in women with EIN. Overall, 47% of women with EIN had EC at final histology. Women who obtained their diagnosis by an endometrial biopsy had 65% risk of EC. The overall risk of sentinel node metastases was 6.3%; none of these women had clear polypoid lesions and five out of six obtained their diagnosis through endometrial biopsy. SLN detection is recommended in women with general endometrial thickening and/or where EIN has been diagnosed by endometrial biopsy, whereas individual risk evaluation is suggested in women with polypoid lesions.

## 1. Introduction

Endometrial intraepithelial neoplasia (EIN) is a premalignant condition of the endometrium. To optimize management, the International Endometrial Collaborative Group introduced this nomenclature in 1999 to distinguish between benign hyperplasia and a precancerous lesion [1,2,3]. At that time, the World Health Organization nomenclature (WHO94) was the terminology of choice, dividing endometrial hyperplasia into four subtypes based on cytology (atypical/nonatypical) and gland architecture (simple/complex) [4]. In 2014, an updated WHO version including only two categories of endometrial hyperplasia was published (hyperplasia without atypia and atypical hyperplasia/EIN) [5,6].

Endometrioid endometrial carcinoma (EC), the most common form of EC, usually develops due to progression from atypical endometrial hyperplasia/EIN. The progression to EC may be unevenly distributed within the endometrium and histologic biopsy results may not be representative. The histopathologic diagnosis of EIN is not clearcut. Despite efforts to standardize the diagnostic criteria for EIN, interobserver variability has been demonstrated. Among expert gynecological pathologists, full agreement occurred in only one-third [7]. As a result, as much as 48.5% of women with a preoperative diagnose of EIN are diagnosed with EC following hysterectomy [8,9,10]. The risk of a concomitant cancer may be higher when the preoperative diagnostic is obtained by endometrial biopsy only [9]. Given the risk of progression, or an undiagnosed EC, the treatment of choice is hysterectomy [11,12]. Gestagen treatment with curettage controls is an alternative in case of a wish to preserve fertility or in highly comorbid women [13,14].

The introduction of pelvic sentinel lymph node (SLN) detection in the surgical treatment of EC has resulted in an increased detection rate of metastatic disease while retaining a low rate of surgical morbidity, which is particularly important in low-risk groups [15,16,17,18]. Whether this is valid even for women with preoperative EIN remains unclear [19,20,21,22].

The primary aim of this study was to determine the risk of concurrent EC in women with a preoperative diagnosis of EIN and the impact of the diagnostic tool used and type of endometrial lesion, i.e., as represented by a perceived general endometrial thickening or polypoid lesion. The secondary aim was to investigate the risk for SLN metastases in women with EC on final histology.

## 2. Materials and Methods

### 2.1. Study Design

This prospective observational study was performed at a tertiary referral center for gynecologic cancer surgery in Sweden (Skåne University Hospital, Lund). A SLN concept has been routinely performed since June 2014 where women with low-risk EC have been offered to take part in a “SLN only” study (clinicalTrials.gov NCT03838055). The FIGO 2009 classification for EC was used [23]. From July 2019 women with EIN were offered inclusion in the study. Data was continuously collected and data on women with preoperative EIN was extracted (Table S1). Preoperative diagnosis was decided on specimen obtained from either an endometrial biopsy, hysteroscopy or dilation and curettage (D&C). All women were examined with vaginal ultrasonography by the designated surgeon to estimate any myometrial invasion. The study was approved by the institutional review board (Skåne University Hospital Dnr 2018/541). A written informed consent was obtained from all enrolled women.

Enrolled women were scheduled for a robotic hysterectomy, bilateral salpingoophorectomy and a pelvic SLN biopsy. As a rule, women underwent surgery 2 to 6 weeks from the initial diagnosis. The SLN procedure was performed according to a previously published anatomically based surgical algorithm using cervically injected indocyanine green (ICG) as tracer [18]. FireFly^®^ mode of the da Vinci^®^ Si or Xi surgical robot (Intuitive Surgical, Sunnyvale, CA, USA) was used to identify the upper paracervical pathway (UPP) including parallel lymphatics to the obturator, the external iliac and the common iliac areas [18]. Reinjection of ICG was performed in case of unilateral or bilateral non-display. Due to an amendment of the SLN protocol based on preliminary data suggesting a correlation between non-mapping of metastatic nodes and non-mapping at defined anatomic positions, non-mapped lymph nodes at those typical positions along the UPP (proximal obturator positions and interiliac positions) were removed and depicted “SLN anatomy” despite other mapped nodes along the UPP [24]. Enlarged, metastatic suspect nodes were removed and labeled “SLN-macro”. The parauterine lymphovascular tissue (PULT) was removed separately as a whole and processed as SLN tissue regardless of mapping [25,26,27,28]. In the case of non-mapping despite reinjection, an anatomically based selective nodal sampling was performed analogue to the “SLN anatomy concept” [29]. In a previous large prospective study evaluating the sensitivity of the SLN-algorithm no isolated SLN metastases were found along the lower paracervical pathway (LPP) in women with FIGO grade 1–2 EC why SLNs along this pathway were not removed further on [18].

The surgery was performed by either one of three initial surgeons. Subsequently, two additional surgeons were gradually introduced; all were initially supervised by the surgeon responsible for study protocol (JP). One of the three initial surgeons was present during all procedures.

### 2.2. Histological Analysis

All SLN-tissue was embedded and bisected or sliced if the minimum thickness exceeded three mm. Ultrastaging using hematoxylin and eosin staining (H&E) was performed in five sections at two to three different levels; 200 µm apart, if the maximum diameter of the sentinel node tissue exceeded 1 mm. Immunohistochemistry (IHC) with staining for pan-cytokeratin (cytokeratin MNF 116) was performed. Non-SLNs with a thickness less than 3 mm were embedded entirely and for nodes exceeding 3 mm at least half the node was embedded. Non-SLNs were stained for H&E but were not subjected to IHC. Metastatic disease was classified according to a modification of the American Joint Committee on Cancer staging definitions for axillary nodes in breast cancer (macro-metastases = tumor greater than 2.0 mm in diameter, micro-metastases = tumor cell aggregates between 0.2 and 2.0 mm in diameter, isolated tumor cells = individual tumor cells or aggregates that are less than 0.2 mm in diameter and less than 200 cells) [30]. For definition of preoperative histologic subtype, we used the initial results based on endometrial biopsy and for definition of the final histologic subtype the complete hysterectomy specimen.

### 2.3. Statistics

Data management was performed using Microsoft^®^ Excel. Statistical analysis was performed using IBM^®^ SPSS^®^ statistics version 29. A *p*-value of <0.05 was considered statistically significant. Differences in baseline characteristics between the two cohorts, final EC and non-EC respectively, were analyzed using Chi-square for categorical variables and Student’s *t*-test or Mann–Whitney U-test for continuous variables. Distribution patterns of differences in age, body mass index and endometrial thickness was visually inspected. Age presented a symmetric distribution; thus, Student’s *t*-test was used.

## 3. Results

**Figure 1:**
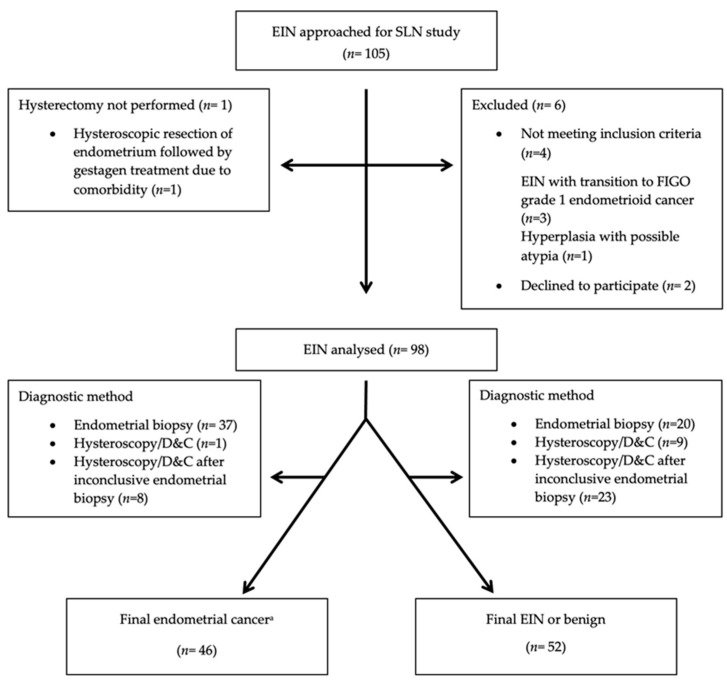
Strobe flow chart of consecutive women with endometrial intraepithelial neoplasia (EIN) assessed for a prospective study evaluating final postsurgical pathology and sentinel lymph node (SLN) status during study period 3 July 2019–16 May 2024. D&C = dilation and curettage. ^a^ Two women did not undergo SLN biopsy due to change in initial approval of SLN removal on the day of surgery and conversion to laparotomy following intraoperative respiratory instability. They were included in the overall study but excluded from the SLN analysis.

A total of 105 consecutive women with EIN were approached for the study, of which 98 were included and data from 96 women were available for the final analysis of SLN data (Table S1, Figure 1). One woman changed her initial approval of a SLN removal on the day of surgery and one woman was converted to laparotomy due to intraoperative respiratory instability. Both had EC on final histology. One woman changed her initial approval of oophorectomy on the day of surgery. She had EC on final histology. Women with preoperative EIN and EC on final histology had a mean age of 70 years compared to 61 years for women in whom the diagnosis remained unchanged (*p* < 0.001), representing the only preoperative parameter associated with a final EC. Demographic and clinical data are presented in Table 1. Separated demographic and clinical data on postoperative EIN and benign diagnoses are presented in Table S2.

The bilateral mapping rate before and after reinjection of ICG was 79% and 86%, respectively. Another 11% of women mapped unilaterally, whereas 3% did not map at all despite reinjection. For the three experienced surgeons, the median time for surgery (skin to skin including docking of the robot) was 70 min (range 42–153 min), of which 18 min (range 8–41 min) were used for detection and removal of SLNs including the PULT. Sub-time for the latter was not separately registered but estimated at 2 minutes per side (Table S3). In four women the vaginal ultrasonography suggested a myometrial invasion. All had a myoinvasive but SLN-negative EC.

In total, 47% (46/98) were diagnosed with EC on final pathology; 42 were classified as FIGO grade 1, three as FIGO grade 2 and one as a carcinosarcoma. In women where the preoperative EIN diagnosis was obtained by an endometrial biopsy, 37/57 (65%) had EC at final histology compared to eight of 31 women (26%) diagnosed by hysteroscopy following an inconclusive endometrial biopsy (*p* < 0.001)). Overall, 74% (23/31) of the latter had polypoid lesions, i.e., in the 28 patients with polypoid lesions endometrial biopsy was only successful in 5 (18%). In case of general endometrial thickening, endometrial biopsy was successful in 50 of 57 women (88%). Hence, a successful endometrial biopsy was more common in cases of general endometrial thickening compared to polypoid lesions (*p* < 0.001).

Out of 46 women with EC 6 (13%), all with non-polypoid lesions, had at least one metastatic SLN, representing a 6.3% overall risk for SLN metastases in women with preoperative EIN and a 9% risk for SLN metastases in women with non-polypoid EIN lesions. Five of these women had a myoinvasive EC at final histology, none of which diagnosed as myoinvasive by preoperative vaginal ultrasonography (Table 2). A paraaortic restaging was performed in two women, both of which had paraaortic metastases. The remaining four were not restaged (SLNs with one ITC only or surgical comorbidity). Detailed data on women with SLN metastases are presented in Table 3.

## 4. Discussion

Overall, 46/98 (47%) of women with EIN had an endometrial cancer on final histology and 13% of these had SLN metastases. The overall risk of SLN metastases in women with preoperative EIN was 6.3%. This corresponds to the risk of SLN metastases (including ITCs) in a large prospective study, taking into account the proportions of women with preoperative EIN and myoinvasiveness of EC on final histology in this study [31]. Overall, 65% diagnosed with EIN through an endometrial biopsy had EC on final histology compared to 26% of women in whom the diagnosis was obtained through hysteroscopic resection and/or D&C following an inconclusive endometrial biopsy and 10% of women in whom a primary hysteroscopy/D&C was performed.

In women diagnosed with EIN, an ultrasonographic perception of a general endometrial thickening was associated with an 88% rate of a conclusive endometrial biopsy compared to a rate of only 18% in women with polypoid lesions. Hence, to individualize care and for logistic and resource reasons we suggest that a hydrosonography should be added to the vaginal ultrasonography when a polypoid lesion is suspected to allocate women to a primary hysteroscopy only when a polypoid lesion is confirmed.

The overall proportion of final EC in our study is concordant with other studies where EC was diagnosed by final histology in 37.7–48.5% of women with a preoperative EIN or atypical hyperplasia [8,10,21,32]. Leitao et al. compared D&C with endometrial biopsy and found that complex atypical hyperplasia (CAH) was upgraded to EC post-hysterectomy in 27% after D&C and in 46% after endometrial biopsy [9]. The difference in definition between CAH and EIN with regards to benign and premalignant lesions may explain the lower overall risk for EC but the results support our finding that a diagnosis of EIN by endometrial biopsy may act as at surrogate marker for generalized hyperplasia and a higher risk for EC [1]. On the other hand, Matanes et al. failed to find a difference in the risk for EC between endometrial biopsy and D&C/hysteroscopy. The type of endometrial lesion or whether D&C/hysteroscopy was the primary or secondary diagnostic method was not specified [21].

Matanes et al. found SLN metastases in 3.3% of EC, which translates to 1.2% of all preoperative EIN, and concluded that SLN status impacted the treatment of 14 out of 162 women with preoperative EIN. Mueller et al. detected positive SLNs in as little as 1.2% of EC and less than 1% of all preoperative EIN [21,32]. In the latter study as little as 2.5% women had a myoinvasive EC. In both studies it is unclear whether, as in our study, ITCs were defined as SLN metastases. The SLN protocol utilized in our study stating exploration of parallel lymphatics along the UPP may further contribute to the discrepancy. In this study, 3/6 node positive women had isolated SLN metastases in the easily overlooked obturator fossa. This corresponds to the results by Bollino et al. where 25.3% of node positive women with EC had isolated obturator metastases [29].

The diagnosis of EIN remains challenging. The interobserver variation is prominent, even among specialist gynecological pathologists [33]. Given the complexity of EIN, we aimed to identify clinical features that may assist in the surgical management of women with a preoperative diagnosis of EIN. The overall risk of SLN metastases of 6.3% in the present study indicates that SLN mapping should be considered in all women with preoperative EIN estimated as a part of general endometrial thickening, i.e., where diagnosis was obtained by endometrial biopsy. In those women, given the known inaccuracy of preoperative myometrial invasion (MI) estimates, regardless of the use of vaginal ultrasonography or MRI, we believe that detection of SLNs should be considered even in women where no deep MI is suspected [34]. No woman with EIN in polypoid lesions diagnosed by hysteroscopy had SLN metastases suggesting that SLN detection might be omitted following an individual evaluation.

The strength of this prospective study is the adherence to a well-established proven high-sensitivity SLN algorithm performed by a limited number of experienced surgeons. Ultrastaging and final histology was performed by expert oncogynecologic pathologists. Furthermore, per guidelines, women with EIN in the southern region of Sweden shall be referred to our gynecologic oncology center. Hence, our results, based on consecutive women, are likely representative for the population. We cannot exclude that a few women with EIN may have undergone surgery at the local hospital, a potential minor weakness. Biomarkers were not routinely applied preoperatively on EIN and postoperatively only in select cases, another weakness of our study.

## 5. Conclusions

A preoperative diagnosis of EIN in women where the diagnosis was obtained by endometrial biopsy was associated with a 65% risk of EC. Of those, 13% had metastatic SLNs. The overall risk of SLN metastases was 6.3% and all metastases were seen in women with a general endometrial thickening or a diagnostic endometrial biopsy, suggesting that SLN detection should be offered particularly to women with EIN who fulfill these preoperative criteria. An individual risk evaluation is recommended in women with polypoid lesions. The complexity of EIN calls for an expert preoperative evaluation, in our opinion at a gynecologic oncology center with a second opinion of histology in select cases, primarily in women with a wish to retain the uterus.

## Figures and Tables

**Table 1:** Clinical characteristics of 98 women with preoperative EIN undergoing hysterectomy and offered sentinel lymph node dissection.

Characteristics Median (Min–Max) or *n* (%) as Appropriate	All Women	Final Pathologic Diagnosis		*p*-Value
		EC	EIN or Benign	
Total	98	46/98 (47%)	52/98 (53%)	
Age (years)	64 (31–86)	70 (44–86)	61 (31–80)	<0.001
BMI (kg/m^2^)	31.4 (19.5–51.9)	29.4 (19.5–51.9)	33.2 (20.9–51.0)	0.123
Parity	2 (0–5)	2 (0–4)	2 (0–5)	0.175
Premenopausal	15/98 (15%)	5/46 (11%)	10/52 (19%)	0.251
ASA				0.408
1–2	82/98 (84%)	40/46 (87%)	42/52 (81%)	
3–4	16/98 (16%)	6/46 (13%)	10/52 (19%)	
BMI ≥ 40 kg/m^2^	13/98 (13%)	5/46 (11%)	8/52 (15%)	
Diagnostic method				<0.001 ^a^
Endometrial biopsy	57/98 (58%)	37/57 (65%)	20/57 (35%)	
Hysteroscopy/D&C	10/98 (10%)	1/10 (10%)	9/10 (90%)	0.001 ^b^
Hysteroscopy/D&C after inconclusive endometrial biopsy	31/98 (32%)	8/31 (26%)	23/31 (74%)	<0.001 ^c^
Endometrial thickness (mm)	14 (3–50)	13 (5–50)	14 (3–33)	0.625
Sonographic evaluation				
MI > 50%	4/98 (4%)	4/4 (100%)	0	
Isolated polyp(s)	34/98 (35%)	8/34 (24%)	26/34 (76%)	0.001 ^d^
General endometrial thickening	61/98 (62%)	35/61 (57%)	26/61 (43%)	
N/A	3/98 (3%)	3/3 (100%)	0	

Data are presented as median (min-max) or absolute number (percentage). EC = endometrial cancer. EIN = endometrial intraepithelial neoplasia. BMI = body mass index. ASA = American Society of Anesthesiologists. D&C = dilation and curettage. MI = myometrial invasion. N/A = not applicable. ^a^ Endometrial biopsy vs. all hysteroscopy/D&C; ^b^ endometrial biopsy vs. primary hysteroscopy/D&C; ^c^ endometrial biopsy vs. hysteroscopy/D&C after inconclusive endometrial biopsy; ^d^ isolated polyp(s) vs. general endometrial thickening.

**Table 2:** Characteristics of 46 women with a preoperative diagnosis of EIN with a final diagnosis of EC.

	EC *n* = 46 Median (Min–Max) or *n* (%) as Appropriate
Histology	
Endometrioid FIGO grade 1	42/46 (91%) (4 SLN+)
Endometrioid FIGO grade 2	3/46 (7%) (2 SLN+)
Carcinosarcoma	1/46 (2%) (0 SLN+)
Diagnostic method	
Endometrial biopsy	37/46 (80%) (5 SLN+)
Hysteroscopy/D&C	1/46 (2%) (1 SLN+)
Hysteroscopy/D&C after inconclusive endometrial biopsy	8/46 (17%) (0 SLN+)
Largest tumor diameter (mm) ^a^	
All	30 (3–100)
FIGO grade 1	30 (3–100)
FIGO grade 2	45 (30–45)
High grade carcinosarcoma	30 (30–30)
Myometrial invasion ≥ 50%	
Total	14/46 (30%) (5 SLN+)
Endometrioid FIGO grade 1	12 ^b^/14 (86%) (3 SLN+)
Endometrioid FIGO grade 2	2/14 (14%) (2 SLN+)
Carcinosarcoma	0 (0 SLN+)
LVSI	
Yes	5/46 (11%) (5 SLN+)
No	41/46 (89%) (1 SLN+)
2009 FIGO stage	
IA	30/46 (65%)
IB	9/46 (20%)
II	1/46 (2%)
IIIC1	4 ^c^/46 (11%)
IIIC2	2 ^d^/46 (2%)
Cancer recurrence ^e^	
Yes	2/46 (4%) (1 SLN+)
No	44/46 (96%) (5 SLN+)

EC = endometrial cancer; ^a^ macroscopic measurement by pathologist. ^b^ One woman also had engagement of cervical stroma; ^c^ one woman had a synchronous endometrioid adenocarcinoma and serous tubal intraepithelial carcinoma in final pathology; ^d^ final stage after surgical restaging; ^e^ up to 18 March 2024.

**Table 3:** Characteristics of six women with preoperative EIN, final endometrial cancer and metastatic sentinel lymph nodes.

	Preoperative		Postoperative								
Case No.	Diagnostic Method	Type of Endometrial Lesion	Surgical Stage	Uterine Stage	MI (%)	Histology	LVSI (Y/N)	SLNs incl. PULT (*n*=) Metastatic/Total	Location and Size of Metastases (incl. SLNs)	Paraaortic Restaging (Y/N)	Cancer Recurrence ^a^ (Y/N)
1	EB	GET	IIIC1	IB	>50	Endometrioid FIGO grade 2	Y	1/4	Obturator L, ITC.	N	Y. Lung metastasis.
2	EB	GET	IIIC2	IA	<50	Endometrioid FIGO grade 1	Y	2/3	Obturator L and R, ITC.	Y. Two LN+. ITC.	N
3	EB	GET	IIIC1	IB	>50	Endometrioid FIGO grade 2	Y	2/3	Extern iliac L and obturator L, ITC.	N	N
4	Hyst	GET	IIIC1	IB	>50	Endometrioid FIGO grade 1	Y	2/5	Obturator L and R, ITC.	N	N
5	EB	N/A	IIIC1	II	>50	Endometrioid FIGO grade 1. 1.2 mm STIC in right fallopian tube	N	1/7	Extern iliac L, ITC.	N	N
6	EB	N/A	IIIC2	IB	>50	Endometrioid FIGO grade 1 w. mucinous differentiation	Y	5/5	Ovary R. PULT R, ITC. Extern iliac L, micro. Obturator L and R, unspecified whether micro or macro. Extern iliac R, ITC.	Y. One LN+. Macro.	N

EIN = endometrial intraepithelial neoplasia, SLN = sentinel lymph node, MI = myometrial invasion, PULT = parauterine lymphovascular tissue, Y = yes, N = no, GET = general endometrial thickening, Hyst = hysteroscopy, N/A = not applicable, ITC = isolated tumor cells, L = left, R = right, STIC = serous tubal intraepithelial carcinoma. ^a^ Up to 18 March 2024.

## Data Availability

All data are available upon request.

## Supplementary Materials

The following supporting information can be downloaded at: https://www.mdpi.com/article/10.3390/cancers16244215/s1, Table S1: Eligibility criteria for sentinel lymph node (SLN) detection in women with endometrial intraepithelial neoplasia (EIN); Table S2: Clinical characteristics of 98 women with preoperative EIN undergoing hysterectomy and offered sentinel lymph node dissection; Table S3: Surgical data on 96 women with EIN undergoing robotic hysterectomy, bilateral salpingoophorectomy and sentinel lymph node (SLN) dissection.
