# Supplementary material for: Endometrial Intraepithelial Neoplasia, Concurrent Endometrial Cancer and Risk for Pelvic Sentinel Node Metastases
Source: Cancers (Basel). 2024 Dec 18;16(24):4215. doi: 10.3390/cancers16244215 (PMC11674175; doi:10.3390/cancers16244215)
Supplement: Supplementary file 1 [file cancers-16-04215-s001.zip › cancers-3290390-supplementary.pdf]

Table S1. Eligibility criteria for sentinel lymph node (SLN) detection in women with endometrial intraepithelial neoplasia (EIN).

**Overall eligibility criteria**	**Inclusion criteria**	**Exclusion criteria**
Age: 18 years and older	Planned robotic operation due to endometrial intraepithelial neoplasia.	No consent.
Sexes: female	Patient suitable for laparoscopic surgery.	Inability to understand study information.
	Signed informed consent.	Surgical or anesthesiologic contraindication for laparoscopic surgery.
		Previous lower limb lymphedema.
		Iodine allergy.
		Disseminated disease.

Table S2. Clinical characteristics of 98 women with preoperative EIN undergoing hysterectomy and offered sentinel lymph node dissection.

**Characteristics Median (min-max) or *n* (%) as appropriate**	**All women**	**Final pathologic diagnosis**		
		**EC**	**EIN**	**Benign/ No remaining lesion**
Total	98	46/98 (47%)	32/98 (33%)	20/98 (20%)
Age (years)	64 (31–86)	70 (44–86)	64 (31–80)	56 (36–73)
BMI (kg/m^2)	31.4 (19.5–51.9)	29.4 (19.5–51.9)	34.3 (21.0–51.0)	28.8 (20.9–40.6)
Parity	2 (0–5)	2 (0–4)	2 (0–5)	2 (0–5)
Premenopausal	15/98 (15%)	5/46 (11%)	6/32 (19%)	4/20 (20%)
ASA				
1–2	82/98 (84%)	40/46 (87%)	23/32 (72%)	19/20 (95%)
3–4	16/98 (16%)	6/46 (13%)	9/32 (28%)	1/20 (5.0%)
BMI≥40 kg/m^2	13/98 (13%)	5/46 (11%)	7/32 (22%)	1/20 (5.0%)
Diagnostic method				
Endometrial biopsy	57/98 (58%)	37/57 (65%)	11/57 (19%)	9/57 (16%)
Hysteroscopy/D&C	10/98 (10%)	1/10 (10%)	7/10 (70%)	2/10 (20%)
Hysteroscopy/D&C after inconclusive endometrial biopsy	31/98 (32%)	8/31 (26%)	14/31 (45%)	9/31 (29%)
Endometrial thickness (mm)	14 (3-50)	13 (5-50)	12 (3-33)	14 (6-28)
Sonographic evaluation				
MI > 50%	4/98 (4%)	4/4 (100%)	0	0
Isolated polyp(s)	34/98 (35%)	8/34 (24%)	17/34 (50%)	9/34 (26%)
General endometrial thickening	61/98 (62%)	35/61 (57%)	15/61 (25%)	11/61 (18%)
N/A	3/98 (3%)	3/3 (100%)	0	0

Data are presented as median (min-max) or absolute number (percentage). EC= endometrial cancer. EIN= endometrial intraepithelial neoplasia. BMI= body mass index. ASA= American Society of Anesthesiologists. D&C=dilation and curettage. MI=myometrial invasion. N/A=not applicable.

Table S3. Surgical data on 96 women with EIN undergoing robotic hysterectomy, bilateral salpingoophorectomy and sentinel lymph node (SLN) dissection.

**Surgical data**<br>**Median (min-max)**	**Experienced surgeon**	**Surgeon under training**
Operative time (min)*	70 (42-153)	100 (50-237)
Time for SLN dissection (min)	18 (8-41)	27 (14-46)
Number SLNs<br>as perceived by surgeon	4 (2-6)	4 (3-6)

*Skin to skin including docking of robot.
